# Sleep and daily pain intensity among Black and White dementia caregivers

**DOI:** 10.1002/alz.71518

**Published:** 2026-05-26

**Authors:** Yee To Ng, Daniel Whibley, Margaret Hicken, Angela Turkelson, Anna Kratz, Kira S. Birditt

**Affiliations:** ^1^ Institute for Social Research University of Michigan Ann Arbor Michigan USA; ^2^ Department of Physical Medicine and Rehabilitation University of Michigan Ann Arbor Michigan USA

**Keywords:** dementia caregiving, ecological momentary assessment, pain experience, racial differences, sleep deprivation

## Abstract

**INTRODUCTION:**

Sleep problems and physical pain are prevalent among dementia caregivers. This study examines daily links between sleep and next‐day pain, focusing on differences between Black and White caregivers.

**METHODS:**

Dementia caregivers (*N *= 210, 34% Black) completed baseline interviews and 5 days of ecological momentary assessments. Each morning, they reported sleep duration and disturbances from the previous night. Pain intensity was reported six times daily and summarized as daily average and time‐specific scores.

**RESULTS:**

Black caregivers reported shorter sleep duration but lower daily pain than White caregivers. In the full sample, greater sleep disturbances predicted higher morning pain. Longer‐than‐usual sleep duration predicted lower next‐day pain (especially evening pain), observed only among Black caregivers.

**DISCUSSION:**

Findings highlight the role of daily sleep patterns in pain. Sleep duration may be a beneficial intervention target for Black caregivers, who reported shorter sleep. Promoting consistent, healthy sleep may help manage pain among dementia caregivers.

## Highlights

- Black caregivers reported shorter sleep and lower daily pain than White caregivers.
- More sleep disturbances were associated with greater morning pain among caregivers.
- Longer sleep was linked to reduced next‐day pain only among Black caregivers.
- Typical sleep patterns, not daily variations, show stronger links to daily pain.

## 1 BACKGROUND

Approximately 7.4 million Americans 65 years of age and older are living with dementia in 2025, and an estimated 12.7 million family members and other unpaid caregivers provide care for people living with this condition.^1^ Among these caregivers, an estimated 50%–74% experience sleep disturbances,^2^ a rate that is significantly higher than non‐dementia caregivers and non‐caregivers.^3^, ^4^, ^5^, ^6^ According to an Alzheimer's Association report,^1^ nearly 40% of dementia caregivers experience physical stress. Other studies indicate that over 60% of dementia caregivers have had musculoskeletal injuries in the past year, with around one‐third reporting back pain and over one‐quarter reporting arthritis.^7^, ^8^ These conditions may stem from physically demanding caregiving tasks such as lifting and mobility support for people living with dementia.

**RESEARCH IN CONTEXT**

1. **Systematic review**: The authors reviewed articles on sleep and pain in the context of dementia caregiving and racial disparities using Google Scholar. They referenced studies highlighting sleep problems and physical pain among dementia caregivers, and research on the role of sleep in pain reduction, particularly those using daily diary or ecological momentary assessment (EMA) methodologies.
2. **Interpretation**: Our EMA results suggest that daily variations in sleep matter: greater sleep disturbances are linked to increased morning pain among dementia caregivers, whereas longer‐than‐usual sleep is linked to reduced next‐day pain, primarily in Black caregivers. Despite these significant daily fluctuations, typical sleep patterns may be more consistently associated with pain in dementia caregiving.
3. **Future directions**: We call for targeted sleep‐based interventions that promote consistent, healthy sleep patterns for pain management among dementia caregivers. Future research should investigate the biopsychosocial factors and mechanisms underlying sleep—pain associations.

Despite the high prevalence of sleep and physical challenges among dementia caregivers, ecological momentary assessment (EMA) studies have focused primarily on psychological outcomes such as depressive symptoms, anxiety, and emotional stress.^9^, ^10^, ^11^ Physical conditions, particularly pain, have received less attention. A recent review identified only eight studies on physical pain in dementia caregivers published in the past decade,^12^ and only one used an EMA design.^13^ Indeed, some dementia caregivers, particularly those with comorbid poor health, may experience greater pain intensity than the general U.S. population with chronic pain, warranting investigation of pain as a critical yet understudied outcome among dementia caregivers.^14^ Although studies have examined the association between sleep and psychological outcomes in dementia caregivers,^15^, ^16^, ^17^, ^18^, ^19^ research has yet to investigate the daily association between sleep and physical pain in this at‐risk population. Focusing on physical pain and identifying daily sleep–pain patterns is crucial, as unaddressed pain can compromise caregiver well‐being and the quality of care, while sleep, as a modifiable behavior, may help alleviate pain.

Conversely, substantial EMA research has shown that poor sleep is linked to heightened pain in healthy and clinical populations (e.g., chronic pain, osteoarthritis).^20^, ^21^, ^22^ However, studies suggest pain is less consistently linked to subsequent sleep quality.^23^, ^24^ Furthermore, clinical research suggests that poor sleep quality most strongly predicts pain in the early morning,^22^, ^25^ highlighting the need to examine time‐of‐day effects among dementia caregivers.

Population studies show that Black individuals generally report shorter, lower‐quality sleep^26^, ^27^, ^28^, ^29^ and higher pain intensity and prevalence of severe pain than White individuals,^30^, ^31^, ^32^ patterns that may reflect racially inequitable social conditions (e.g., socioeconomic disadvantage, cumulative social stressors, environmental exposures, and limited access to health care). However, research on racial differences in sleep and pain outcomes among dementia caregivers is limited and it remains unclear whether the sleep–pain link varies by race. Sleep may serve as an important coping resource, particularly under challenging circumstances.^33^ Because Black caregivers may experience greater exposure to chronic challenges, they may rely more on sleep for physical restoration, pain regulation, and resilience; therefore, better sleep may be more strongly linked to reduced pain compared to White caregivers.^34^, ^35^ Notably, any differences observed between Black and White caregivers are interpreted as reflecting socially patterned experiences associated with caregivers’ racial group, rather than inherent biological differences.

Using EMA methodology, we primarily examined within‐person associations between sleep and next‐day pain among Black and White dementia caregivers, while also assessing between‐person associations to provide a more complete picture. This study addresses:

1. Are there racial differences in daily sleep and pain intensity?
*H1*: White caregivers will report better daily sleep (i.e., longer duration, fewer disturbances) and lower levels of pain intensity than Black caregivers.
2. Does sleep predict next‐day pain intensity, and does this vary by time of day?
*H2*: Better sleep will be associated with lower next‐day pain, and more strongly associated with reduced morning pain than with afternoon or evening pain.
3. Do the associations between sleep and pain intensity differ by race?
*H3*: The association between sleep and pain will be stronger for Black caregivers than White caregivers.

## 2 METHODS

### 2.1 Sample and procedures

This study used data from the Stress and Well‐Being in the Everyday Lives of Caregivers Study (SWELCare), conducted in the Midwest region of the United States including Michigan and Ohio between December 2021 and August 2024.^36^ The study included 247 adults who provided unpaid care to a *co‐residing* adult family member or friend with dementia. To be eligible, caregivers had to be the primary caregiver, report that their care recipients had at least two items on the AD8 Dementia Screening Interview (a brief questionnaire designed to detect signs of dementia),^37^ identify as Black or White, and be able to speak and read English.

Caregivers first completed a 90–120 min baseline phone interview covering demographics, health conditions, and caregiving situations, and then 5 days of EMAs. Caregivers completed EMA surveys on study‐provided phones six times daily (upon waking, at 9 a.m., 12 p.m., 3 p.m., and 6 p.m., and before bed) over 5 days. Surveys upon waking assessed the previous night's sleep, whereas pain intensity was reported at each survey time. The final analytic sample included 210 caregivers (85%) who completed the baseline interview, participated in the EMA, and provided at least one valid sleep measure from the morning surveys. Exclusions were due to non‐participation in EMA (*n* = 24) or no sleep data (*n* = 13). The final sample did not differ from the 37 excluded participants in terms of sociodemographics, health conditions, or caregiving situations.

Participants were compensated up to $340, including $50 for the baseline interview, $50 per day for the 5 days of EMA, and a $40 bonus for completing all parts of the study. The study protocol was approved by the University of Michigan Ethics Review Board, and all participants provided informed consent.

### 2.2 Ecological momentary assessment (EMA)

This study focuses on two dimensions of sleep: sleep duration and levels of sleep disturbance.

#### 2.2.1 Sleep duration

Upon waking, participants reported their sleep duration from the previous night in response to the prompt: “*How many hours of sleep did you get last night? This may be different from the number of hours you spent in bed*.” Responses were recorded in solid hours and 15 min increments. A continuous variable representing total sleep duration (in hours) was then calculated.

#### 2.2.2 Levels of sleep disturbance

Every morning (i.e., on the first survey of each day), sleep disturbance was assessed using a modified Jenkins Sleep Scale^38^ with four yes/no items: “*Did you have trouble falling asleep*?”, “*Did you wake during the night and struggle to fall back asleep*?”, “*Did you wake too early*?”, and “*Do you feel unrested this morning*?”. Consistent with prior use in the literature, responses were summed,^39^, ^40^, ^41^ with higher scores indicating greater levels of sleep disturbance each night.

#### 2.2.3 Pain intensity

Caregivers’ evaluation of pain may vary across the day in response to caregiving demands, such as in the late afternoon or evening when care recipients may experience sundowning; therefore, the EMA design of this study assessed pain six times daily. The wake‐up survey asked, “*How much physical pain or discomfort are you currently experiencing?*” Later EMA surveys asked, “*In the last 3 h, how much physical pain did you experience?*” Responses were rated on a 6‐point scale 1 (*none*), 2 (*very mild*), 3 (*mild*), 4 (*moderate*), 5 (*severe*), and 6 (*very severe*). These responses were recoded to range from 0 (*none*) to 5 (*very severe*). We computed *average daily pain* scores by taking the mean across all six assessments to reflect *overall pain* for that day. Time‐specific pain scores were also calculated: morning pain (average pain scores of wake‐up, 9 a.m., and 12 noon surveys), afternoon pain (average pain scores of 3 p.m. and 6 p.m. surveys), and evening pain (pain level reported at bedtime). Each of these pain outcomes was modeled separately as day‐level outcomes.^22^

### 2.3 Day‐level covariates

We controlled for nighttime caregiving, assessed with the question: “*Did you provide care assistance to PLWD* [person living with dementia] *during* *your regular sleeping hours last night?*” coded as 1 (*yes*) and 0 (*no*).

### 2.4 Baseline interview variables

#### 2.4.1 Race

Self‐reported race was coded as 1 (*Black*) and 0 (*White*).

#### 2.4.2 Participant‐level covariates

We adjusted for several covariates collected during the baseline interview, including: **caregivers’ sociodemographics**: age (continuous), gender (1 *female*, 0 *male*), marital status (1 *married/cohabitated*, 0 *not married*), education (1 *college or above degree*, 0 *less than college*), employment (1 *work part‐or full‐time*, 0 *not working*); **caregivers’ health conditions**: depressive symptoms (sum score of the 8‐item Center for Epidemiologic Studies Depression Scale [CES‐D])^42^ and anxiety (average of four‐items: nervous, tense, afraid, worrying),^43^ and body max index (BMI; calculated by dividing an individual's weight in kilograms by the square of their height in meters); and **caregiving situations**: caregiver relationship to the care recipient was dummy coded as spouse 1 (*yes*) 0 (*no*), adult child 1 (*yes*) 0 (*no*), or other caregiver 1 (*yes*) 0 (*no*). Caregiver burden was measured using the 12‐item Zarit Burden Scale^44^, ^45^ (possible range: 0–48), with higher scores indicating greater burden. Years of caregiving was included as a continuous variable.

### 2.5 Analytic strategy

Descriptive statistics, both full sample and stratified by race, were computed at the participant level to examine racial differences using *t*‐tests or chi‐square tests. Bivariate correlations at the between‐person level (*Level 2*) were also conducted to assess associations among key variables. To illustrate how sleep and pain levels varied throughout the study period, we plotted mean scores with 95% confidence intervals (CIs) across five study days and six time points: upon waking; at 9 a.m., noon, 3 p.m., and 6 p.m.; and before bed. Values were averaged across all available days of data collection. The plots were stratified by race (White vs Black caregivers) to visualize the potential differences in daily patterns.

We estimated multilevel models with days (*Level 1*) nested within participants (*Level 2*). Intraclass correlation coefficient (ICC) indicated that 68% of the variance in pain was due to differences between caregivers (*Level 2*) and 32% was due to within‐person fluctuations (*Level 1*). Our approach focuses on key variance components (within‐person and between‐person) and how sleep affects pain at different times of day (morning, afternoon, and evening; following the procedure used in Tang et al.).^22^

First, to examine racial differences in daily sleep and pain, we estimated multilevel linear models treating race (*Level 2*) as a predictor and daily sleep and daily overall pain (*Level 1*) as separate outcomes. Next, to test whether sleep is associated with next‐day overall pain, we treated sleep as a time‐varying predictor. To isolate within‐person effects (e.g., whether caregivers experience more or less pain on days when they sleep more than their own average), we group‐mean centered the sleep variable by adjusting for each participant's average sleep duration.^46^ This also allowed us to account for between‐person effects (e.g., whether caregivers who typically sleep more during the study period tend to experience less pain overall). We first modeled daily overall pain as the outcome, followed by models examining time‐specific pain indices (morning, afternoon, and evening pain) to test whether sleep is linked to pain differently at different times of the following day. Finally, to examine racial differences in these associations, we added cross‐level interaction terms between race (*Level 2*) and sleep (*Level 1*) to the models. When significant interactions emerged, we conducted simple slope analyses to probe these effects.

All models adjusted for participant‐level covariates and primarily focused on within‐person effects. All analyses were conducted in STATA 18 MP‐2. We used the MIXED command for continuous outcomes. Models were fitted using Restricted Maximum Likelihood (REML) and included random intercepts for each individual caregiver. Random slopes were excluded, as they did not improve model fit, indicating consistent sleep effects across individuals. An unstructured covariance matrix (cov(unstructured)) was specified to flexibly model correlations between random effects. Standard errors were adjusted for within‐subject correlations and cluster‐level heteroskedasticity using vce (cluster id).

## 3 RESULTS

A total of 210 Black and White caregivers contributed 856 morning surveys that included at least one valid sleep measure (*mean* = 4.08 morning surveys per participant, *SD* = 1.23, range = 1–7; 98% provided up to 5 days). Notably, five participants completed more than 5 days of morning surveys because of continued engagement with the EMA prompts beyond the required period, as well as issues with participants receiving EMA surveys. On average, caregivers provided 26.39 EMA surveys (standard deviation [SD] = 5.10; range = 3–47). Table 1 summarizes caregivers’ demographic characteristics, as well as characteristics of their daily sleep and pain intensity. Independent samples *t*‐tests indicated that, compared to White caregivers, Black caregivers had a higher BMI (*t *= 2.84, *p* = 0.005), reported fewer hours of sleep (*t* = 3.63, *p* < 0.001), and provided more nights of care for PLWD *(t* = ‐2.05, *p* = 0.04). The chi‐square tests further showed that Black caregivers were less likely to be married (*χ^2^* = 22.81, *p* < 0.001) or to be spousal caregivers (*χ^2^ *= 29.13, *p* < 0.001) than White caregivers. Bivariate correlations are presented in Table S1 (participant‐level correlations) and Table S2 (assessment‐level correlations). Notably, there was a moderate negative correlation between sleep hours and levels of sleep disturbance at the day level (*r* = –0.44, *p* < 0.001).

**TABLE 1:** Sample characteristics.

	Full sample (*n *= 210)				Black caregivers (*n *= 72)		White caregivers (*n *= 138)	
	*Mean/Prop*.	*SD*	Min.	Max.	*Mean/Prop*.	*SD*	*Mean/Prop*.	*SD*
**Participant characteristics**								
Age, years	61.54	13.26	21.00	87.00	60.11	13.33	62.29	13.22
Female	0.78				0.85		0.75	
Married	0.62				0.40		0.74	
College or above	0.63				0.58		0.65	
Work part‐/full‐time	0.39				0.39		0.39	
Depression	7.20	5.12	0.00	23.00	7.94	5.50	6.81	4.88
Anxiety	2.00	0.66	1.00	4.00	2.11	0.77	1.95	0.60
BMI	30.33	7.49	17.36	55.78	32.33	8.39	29.29	6.78
Caregiving relationship								
Spousal caregivers	0.53				0.28		0.67	
Adult child caregivers	0.35				0.56		0.24	
Other caregivers	0.12				0.17		0.09	
Caregiver burden	20.27	8.62	2.00	46.00	19.81	9.64	20.51	8.05
Help duration (in years)	4.64	4.20	0.08	29.00	4.96	3.99	4.47	4.31
**Day‐level characteristics**								
Sleep hours	6.51	1.24	3.00	9.67	6.09	1.36	6.73	1.11
Sleep disturbances	1.33	1.01	0.00	4.00	1.34	1.11	1.32	0.95
Sleep disturbances (binary)	0.65	0.35	0.00	1.00	0.63	0.37	0.66	0.34
Nighttime caregiving (binary)	0.23	0.33	0.00	1.00	0.29	0.36	0.19	0.32
Overall daily pain	0.91	0.92	0.00	4.00	0.82	0.95	0.96	0.90
Morning pain	0.88	0.89	0.00	4.00	0.80	0.93	0.92	0.87
Afternoon pain	0.93	0.99	0.00	4.25	0.84	1.04	0.97	0.97
Evening pain	0.98	1.05	0.00	4.00	0.86	1.13	1.04	1.01

**FIGURE 1:**
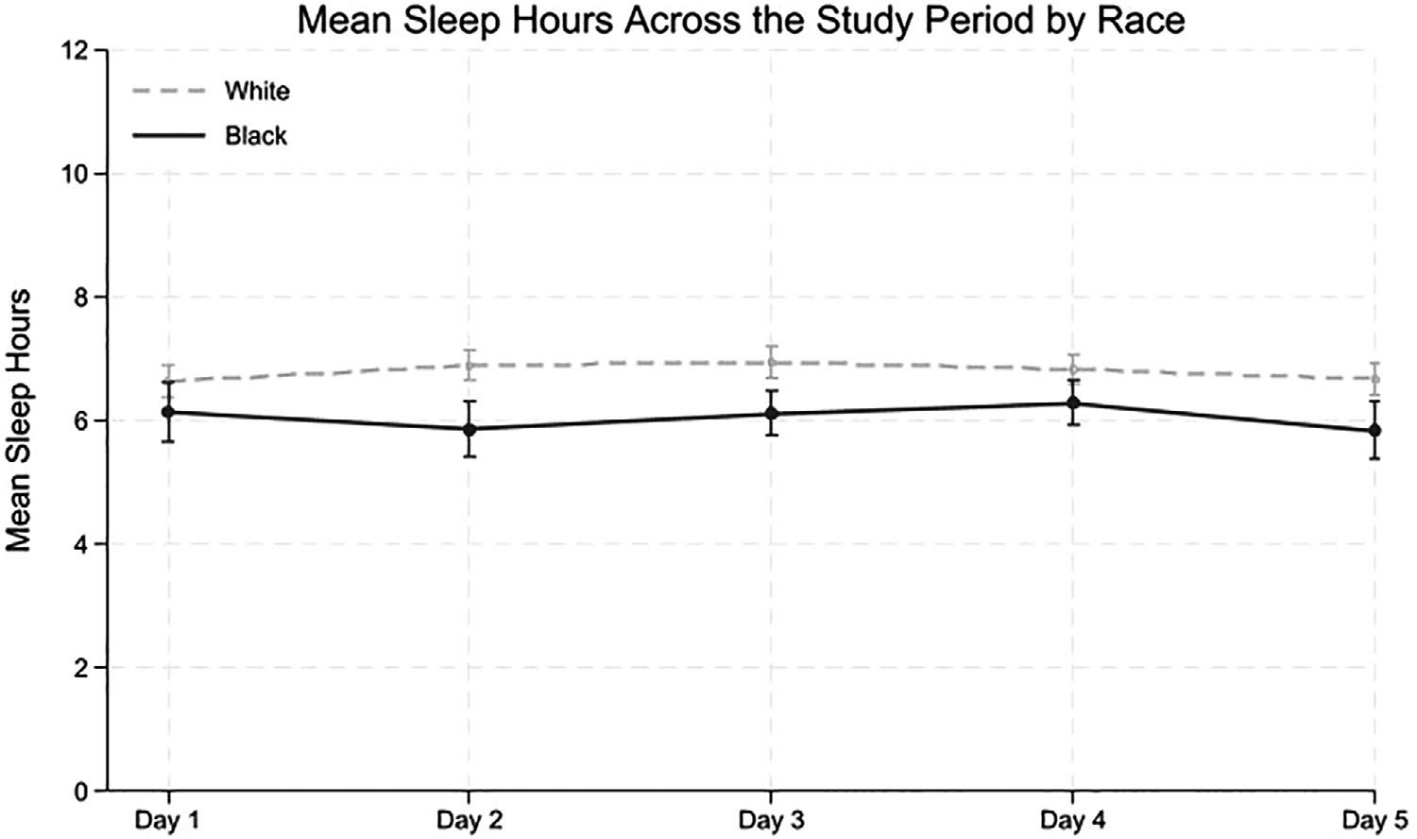
Mean sleep hours across study days by race. *Note*: The figure shows the average sleep hours for each study day across all participants.

**FIGURE 2:**
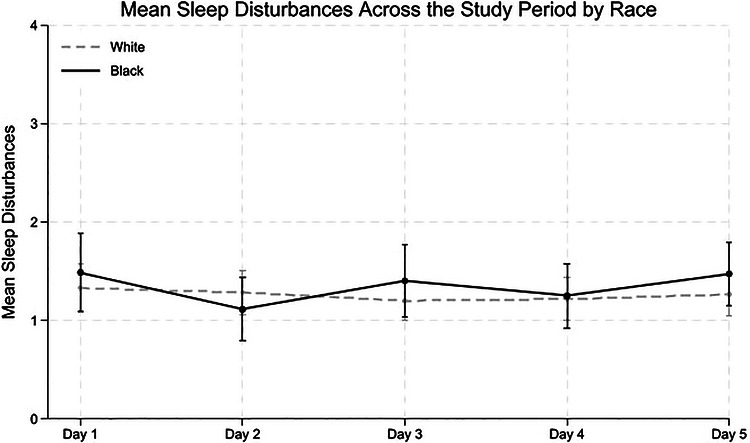
Mean sleep disturbances across study days by race. *Note*: The figure shows the average sleep disturbance for each study day across all participants. Although this figure may visually suggest racial differences in variability across days, multilevel modeling analyses suggest no racial differences in variability (i.e., standard deviation).

**FIGURE 3:**
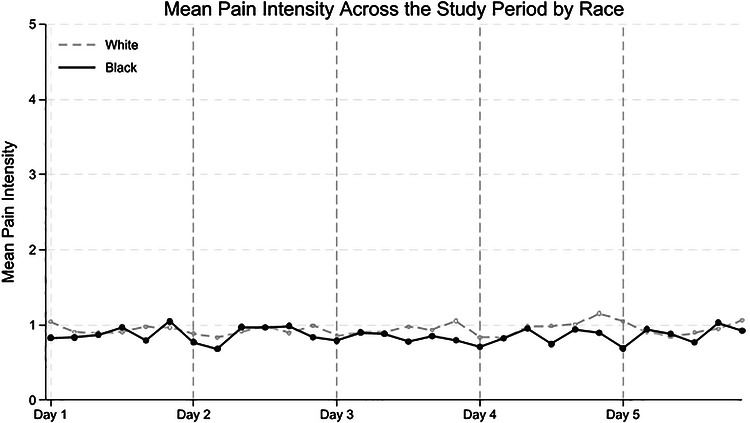
Mean pain intensity across study days by race. *Note*: The figure shows the mean pain intensity for each assessment on each study day across all participants. Although this figure may visually suggest racial differences in variability across days, multilevel modeling analyses suggest no racial differences in variability (i.e., standard deviation).

Figures 1 and 2 shows mean sleep hours and mean levels of sleep disturbance across study days by race. Figure 3 displays mean pain intensity across study days by race, and Figure S1 shows mean pain intensity at different time points of the day.

### 3.1 Racial differences in daily sleep and overall pain

We first examine the differences in sleep duration and disturbances, as well as pain intensity, between racial groups. As shown in Table 2, Black caregivers reported fewer hours of sleep than White caregivers (*B *= ‐0.38, 95% CI: −0.73 to −0.04, *p* = 0.03), even accounting for whether they provide care for the PLWD during the night. There were no significant racial differences in the levels of sleep disturbance (*B* = −0.23, 95% CI: −0.48 to 0.03, *p* = 0.08). In terms of pain, Black caregivers reported significantly lower overall daily pain than White caregivers (*B *= −0.29, 95% CI: −0.55 to −0.03, *p* = 0.03).

**TABLE 2:** Multilevel linear models predicting daily sleep and pain intensity from race.

	Sleep hours			Sleep disturbances			Pain intensity		
	*B (SE)*	*95% CI*	*p*‐value	*B (SE)*	*95% CI*	*p*‐value	*B (SE)*	*95% CI*	*p*‐value
Intercept	6.22 (0.84)	[4.57, 7.87]	0.000	1.25 (0.59)	[0.09, 2.42]	0.035	0.71 (0.62)	[−0.51, 1.93]	0.255
Black	**−0.38 (0.18)**	**[−0.73, −0.04]**	**0.031**	−0.23 (0.13)	[−0.48, 0.03]	0.080	**−0.29 (0.13)**	**[−0.55, −0.03]**	**0.026**
**Participant‐level covariates**									
Age	0.00 (0.01)	[−0.01, 0.02]	0.681	−0.01 (0.01)	[−0.02, 0.01]	0.313	−0.01 (0.01)	[−0.02, 0.01]	0.257
Female	0.40 (0.16)	[0.09, 0.71]	0.012	−0.22 (0.13)	[−0.47, 0.03]	0.090	0.13 (0.15)	[−0.16, 0.42]	0.385
Married	0.15 (0.26)	[−0.35, 0.65]	0.546	−0.01 (0.18)	[−0.36, 0.34]	0.946	0.33 (0.20)	[−0.07, 0.73]	0.106
College or above	0.34 (0.15)	[0.04, 0.64]	0.027	−0.27 (0.12)	[−0.50, −0.03]	0.026	−0.28 (0.13)	[−0.54, −0.02]	0.035
Work part‐/full‐time	−0.10 (0.16)	[−0.42, 0.22]	0.545	0.01 (0.12)	[−0.23, 0.24]	0.951	−0.38 (0.12)	[−0.61, −0.14]	0.002
Depression	−0.05 (0.02)	[−0.09, −0.01]	0.009	0.08 (0.01)	[0.05, 0.11]	0.000	0.06 (0.02)	[0.02, 0.09]	0.001
Anxiety	−0.08 (0.15)	[−0.38, 0.21]	0.580	0.07 (0.11)	[−0.15, 0.30]	0.527	−0.18 (0.13)	[−0.42, 0.07]	0.151
BMI	0.01 (0.01)	[−0.01, 0.03]	0.263	−0.01 (0.01)	[−0.02, 0.01]	0.237	0.02 (0.01)	[0.00, 0.04]	0.052
Spouse caregivers		(REF)			(REF)			(REF)	
Adult child caregivers	−0.32 (0.24)	[−0.80, 0.15]	0.184	0.23 (0.21)	[−0.18, 0.64]	0.270	0.31 (0.22)	[−0.13, 0.75]	0.170
Other caregivers	0.15 (0.32)	[−0.48, 0.77]	0.647	0.21 (0.22)	[−0.23, 0.64]	0.355	0.21 (0.30)	[−0.38, 0.79]	0.493
Caregiver burden	0.00 (0.01)	[−0.01, 0.02]	0.652	0.00 (0.01)	[−0.01, 0.02]	0.627	0.00 (0.01)	[−0.02, 0.02]	0.944
Help duration	0.01 (0.01)	[−0.02, 0.03]	0.707	0.01 (0.01)	[−0.02, 0.03]	0.542	0.00 (0.01)	[−0.02, 0.03]	0.923
**Day‐level covariates**									
Nighttime caregiving (Within‐person)	−0.38 (0.17)	[−0.72, −0.04]	0.030	0.28 (0.13)	[0.02, 0.54]	0.033	0.01 (0.05)	[−0.09, 0.11]	0.829
Nighttime caregiving (Between‐person)	−1.04 (0.26)	[−1.55, −0.54]	0.000	0.64 (0.17)	[0.32, 0.96]	0.000	0.16 (0.18)	[−0.21, 0.52]	0.398
Observations		854			855			855	
No. participants		210			210			210	

### 3.2 Sleep as a predictor of next‐day pain: overall pain and pain at different times of the day

As shown in Table 3, within‐person variations in sleep duration were not significantly associated with overall next‐day pain (*B *= −0.01, 95% CI: −0.03 to 0.02, *p* = 0.72), or morning pain (*B* = −0.00, 95% CI: −0.03 to 0.03, *p *= 0.99), afternoon pain (*B *= −0.01, 95% CI: −0.06 to 0.03, *p* = 0.57), or evening pain (*B *= −0.01, 95% CI: −0.06 to 0.03, *p* = 0.58).

**TABLE 3:** Multilevel linear models predicting overall pain and pain in different times of the day from sleep duration.

	Overall Pain			Morning Pain			Afternoon Pain			Evening Pain		
	*B (SE)*	*95% CI*	*p*‐value	*B (SE)*	*95% CI*	*p*‐value	*B (SE)*	*95% CI*	*p*‐value	*B (SE)*	*95% CI*	*p*‐value
Intercept	1.67 (0.70)	[0.29, 3.05]	0.017	1.63 (0.68)	[0.30, 2.97]	0.016	2.14 (0.77)	[0.62, 3.65]	0.006	1.87 (0.79)	[0.32, 3.42]	0.018
**Within‐person (WP) association**												
Sleep hours	−0.01 (0.01)	[−0.03, 0.02]	0.718	0.00 (0.02)	[−0.03, 0.03]	0.990	−0.01 (0.02)	[−0.06, 0.03]	0.568	−0.01 (0.02)	[−0.06, 0.03]	0.578
**Between‐person (BP) association**												
Sleep hours	**−0.16 (0.05)**	**[−0.26, −0.05]**	**0.003**	**−0.15 (0.05)**	**[−0.25, −0.05]**	**0.004**	**−0.20 (0.06)**	**[−0.32, −0.08]**	**0.001**	**−0.17 (0.06)**	**[−0.29, −0.04]**	**0.010**
**Participant‐level covariates**												
Black	−0.35 (0.13)	[−0.60, −0.09]	0.008	−0.30 (0.13)	[−0.56, −0.04]	0.022	−0.38 (0.14)	[−0.67, −0.10]	0.008	−0.44 (0.14)	[−0.72, −0.16]	0.002
Age	−0.01 (0.01)	[−0.02, 0.01]	0.309	−0.01 (0.01)	[−0.02, 0.01]	0.338	−0.01 (0.01)	[−0.02, 0.01]	0.358	−0.01 (0.01)	[−0.02, 0.00]	0.185
Female	0.20 (0.15)	[−0.09, 0.49]	0.181	0.20 (0.15)	[−0.08, 0.49]	0.165	0.21 (0.16)	[−0.10, 0.52]	0.186	0.16 (0.16)	[−0.15, 0.47]	0.316
Married	0.36 (0.20)	[−0.04, 0.75]	0.078	0.30 (0.19)	[−0.08, 0.68]	0.127	0.32 (0.21)	[−0.10, 0.73]	0.132	0.49 (0.23)	[0.05, 0.94]	0.029
College or above	−0.22 (0.13)	[−0.48, 0.04]	0.091	−0.16 (0.13)	[−0.41, 0.09]	0.217	−0.34 (0.14)	[−0.61, −0.06]	0.016	−0.23 (0.15)	[−0.52, 0.06]	0.127
Work part‐/full‐time	−0.39 (0.12)	[−0.63, −0.16]	0.001	−0.36 (0.12)	[−0.59, −0.13]	0.002	−0.37 (0.13)	[−0.63, −0.11]	0.005	−0.49 (0.14)	[−0.76, −0.22]	0.000
Depression	0.05 (0.02)	[0.02, 0.08]	0.004	0.04 (0.02)	[0.01, 0.08]	0.010	0.05 (0.02)	[0.02, 0.09]	0.005	0.06 (0.02)	[0.03, 0.10]	0.001
Anxiety	−0.20 (0.12)	[−0.44, 0.05]	0.111	−0.25 (0.12)	[−0.49, −0.01]	0.041	−0.18 (0.13)	[−0.44, 0.08]	0.185	−0.15 (0.14)	[−0.42, 0.13]	0.297
BMI	0.02 (0.01)	[0.00, 0.04]	0.026	0.02 (0.01)	[0.00, 0.04]	0.016	0.02 (0.01)	[0.00, 0.04]	0.083	0.02 (0.01)	[0.00, 0.04]	0.047
Spouse caregivers		(REF)			(REF)			(REF)			(REF)	
Adult child caregivers	0.26 (0.22)	[−0.17, 0.69]	0.243	0.15 (0.22)	[−0.28, 0.57]	0.492	0.28 (0.22)	[−0.16, 0.72]	0.212	0.43 (0.25)	[−0.05, 0.92]	0.080
Other caregivers	0.23 (0.30)	[−0.35, 0.82]	0.432	0.13 (0.28)	[−0.41, 0.68]	0.630	0.27 (0.32)	[−0.36, 0.89]	0.402	0.34 (0.35)	[−0.33, 1.02]	0.320
Caregiver burden	0.00 (0.01)	[−0.02, 0.02]	0.960	0.00 (0.01)	[−0.02, 0.02]	0.769	0.00 (0.01)	[−0.02, 0.02]	0.906	−0.01 (0.01)	[−0.03, 0.01]	0.508
Help duration	0.00 (0.01)	[−0.02, 0.03]	0.859	0.00 (0.01)	[−0.02, 0.02]	0.958	0.00 (0.02)	[−0.03, 0.03]	0.772	0.01 (0.02)	[−0.03, 0.04]	0.694
**Day‐level covariates**												
Nighttime caregiving (WP)	0.01 (0.05)	[−0.09, 0.11]	0.859	−0.02 (0.05)	[−0.11, 0.08]	0.731	0.09 (0.07)	[−0.05, 0.23]	0.194	−0.01 (0.12)	[−0.23, 0.22]	0.958
Nighttime caregiving (BP)	−0.01 (0.18)	[−0.36, 0.34]	0.966	0.01 (0.18)	[−0.34, 0.35]	0.974	−0.12 (0.19)	[−0.50, 0.26]	0.533	0.00 (0.21)	[−0.41, 0.41]	0.986
Observations		854			854			830			789	
No. participants		209			209			205			203	

Similarly, as shown in Table 4, within‐person variations in levels of sleep disturbance were not significantly associated with overall next‐day pain (*B* = 0.02, 95% CI: −0.01 to 0.06, *p* = 0.20) or with afternoon pain (*B* = −0.00, 95% CI: −0.06 to 0.05 *p *= 0.97) or evening pain (*B *= −0.02, 95% CI: −0.08 to 0.04, *p* = 0.47). Yet, they were significantly associated with greater morning pain (*B* = 0.05, 95% CI: 0.01−0.09, *p *= 0.02).

**TABLE 4:** Multilevel linear models predicting overall pain and pain in different times of the day from sleep disturbances.

	Overall pain			Morning pain			Afternoon pain			Evening pain		
	*B (SE)*	*95% CI*	*p*‐value	*B (SE)*	*95% CI*	*p*‐value	*B (SE)*	*95% CI*	*p*‐value	*B (SE)*	*95% CI*	*p*‐value
Intercept	0.43 (0.63)	[−0.79, 1.66]	0.487	0.44 (0.60)	[−0.73, 1.61]	0.461	0.60 (0.68)	[−0.72, 1.93]	0.372	0.57 (0.71)	[−0.82, 1.96]	0.425
**Within‐person (WP) association**												
Sleep disturbances	0.02 (0.02)	[−0.01, 0.06]	0.197	**0.05 (0.02)**	**[0.01, 0.09]**	**0.016**	0.00 (0.03)	[−0.06, 0.05]	0.974	−0.02 (0.03)	[−0.08, 0.04]	0.468
**Between‐person (BP) association**												
Sleep disturbances	**0.21 (0.07)**	**[0.06, 0.35]**	**0.005**	**0.22 (0.07)**	**[0.08, 0.35]**	**0.002**	**0.22 (0.08)**	**[0.07, 0.37]**	**0.005**	**0.24 (0.09)**	**[0.07, 0.42]**	**0.006**
**Participant‐level covariates**												
Black	−0.24 (0.13)	[−0.50, 0.01]	0.064	−0.20 (0.13)	[−0.46, 0.07]	0.142	−0.26 (0.15)	[−0.55, 0.03]	0.077	−0.32 (0.14)	[−0.60, −0.04]	0.024
Age	−0.01 (0.01)	[−0.02, 0.01]	0.340	−0.01 (0.01)	[−0.02, 0.01]	0.344	−0.01 (0.01)	[−0.02, 0.01]	0.428	−0.01 (0.01)	[−0.02, 0.00]	0.197
Female	0.18 (0.15)	[−0.10, 0.47]	0.210	0.21 (0.15)	[−0.08, 0.49]	0.156	0.17 (0.16)	[−0.14, 0.48]	0.278	0.14 (0.15)	[−0.16, 0.45]	0.355
Married	0.34 (0.20)	[−0.05, 0.74]	0.087	0.28 (0.19)	[−0.09, 0.66]	0.140	0.30 (0.21)	[−0.11, 0.71]	0.153	0.47 (0.23)	[0.03, 0.91]	0.037
College or above	−0.22 (0.13)	[−0.47, 0.03]	0.080	−0.14 (0.12)	[−0.38, 0.10]	0.245	−0.36 (0.14)	[−0.63, −0.08]	0.011	−0.22 (0.15)	[−0.50, 0.07]	0.135
Work part‐/full‐time	−0.38 (0.12)	[−0.62, −0.15]	0.001	−0.35 (0.11)	[−0.57, −0.13]	0.002	−0.35 (0.13)	[−0.61, −0.10]	0.007	−0.48 (0.14)	[−0.75, −0.21]	0.000
Depression	0.04 (0.02)	[0.01, 0.08]	0.023	0.03 (0.02)	[0.00, 0.07]	0.054	0.04 (0.02)	[0.01, 0.08]	0.023	0.05 (0.02)	[0.01, 0.09]	0.010
Anxiety	−0.19 (0.12)	[−0.43, 0.05]	0.121	−0.24 (0.12)	[−0.48, −0.01]	0.041	−0.17 (0.13)	[−0.43, 0.09]	0.209	−0.15 (0.14)	[−0.43, 0.12]	0.273
BMI	0.02 (0.01)	[0.00, 0.04]	0.025	0.02 (0.01)	[0.00, 0.04]	0.012	0.02 (0.01)	[0.00, 0.03]	0.099	0.02 (0.01)	[0.00, 0.04]	0.036
Spouse caregivers		(REF)			(REF)			(REF)			(REF)	
Adult child caregivers	0.26 (0.22)	[−0.17, 0.69]	0.240	0.14 (0.21)	[−0.28, 0.56]	0.516	0.30 (0.23)	[−0.14, 0.74]	0.186	0.42 (0.25)	[−0.07, 0.91]	0.094
Other caregivers	0.16 (0.30)	[−0.42, 0.74]	0.580	0.06 (0.28)	[−0.48, 0.61]	0.821	0.19 (0.32)	[−0.44, 0.81]	0.556	0.25 (0.34)	[−0.42, 0.92]	0.463
Caregiver burden	0.00 (0.01)	[−0.02, 0.02]	0.855	0.00 (0.01)	[−0.02, 0.02]	0.907	0.00 (0.01)	[−0.02, 0.02]	0.703	−0.01 (0.01)	[−0.03, 0.01]	0.395
Help duration	0.00 (0.01)	[−0.02, 0.02]	0.988	0.00 (0.01)	[−0.02, 0.02]	0.921	0.00 (0.01)	[−0.03, 0.03]	0.907	0.00 (0.02)	[−0.03, 0.03]	0.828
**Day‐level covariates**												
Nighttime caregiving (WP)	0.00 (0.05)	[−0.10, 0.10]	0.941	−0.03 (0.05)	[−0.13, 0.07]	0.545	0.10 (0.07)	[−0.05, 0.24]	0.180	0.01 (0.12)	[−0.22, 0.23]	0.952
Nighttime caregiving (BP)	0.04 (0.19)	[−0.33, 0.41]	0.835	0.04 (0.18)	[−0.32, 0.40]	0.827	−0.04 (0.21)	[−0.44, 0.36]	0.845	0.03 (0.22)	[−0.41, 0.46]	0.907
Observations		855			855			831			790	
No. participants		210			210			206			204	

In contrast, *between‐person differences* in longer sleep duration predicted lower overall pain (*B* = −0.16, 95% CI: −0.26 to −0.05, *p* = 0.003), morning pain (*B* = −0.15, 95% CI: −0.25 to −0.05, *p* = 0.004), afternoon pain (*B* = −0.20, 95% CI: −0.32 to −0.08, *p* = 0.001), and evening pain (*B* = −0.17, 95% CI: −0.29 to −0.04, *p* = 0.01; Table 3). Similarly, between‐person differences in greater levels of sleep disturbance significantly predicted greater overall pain (*B* = 0.21, 95% CI: 0.06−0.35, *p* = 0.005), morning pain (*B *= 0.22, 95% CI: 0.08−0.35, *p* = 0.002), afternoon pain (*B *= 0.22, 95% CI: 0.07−0.37, *p* = 0.005), and evening pain (*B* = 0.24, 95% CI: 0.07−0.42, *p* = 0.006; Table 4).

### 3.3 Racial differences in the sleep−pain associations

**FIGURE 4:**
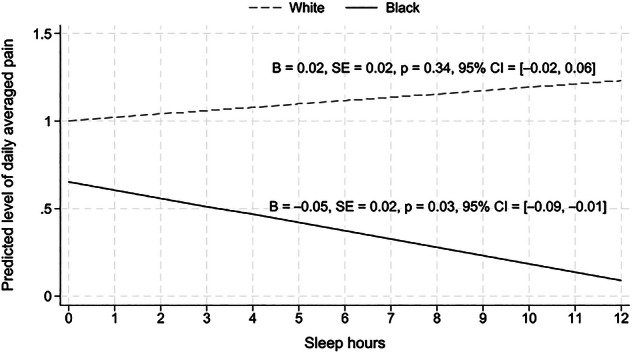
**Within‐person links between sleep duration and daily pain**. Simple slope analysis revealed that, for Black caregivers, sleeping longer than their own average was significantly associated with lower next‐day overall pain, whereas for White individuals, the effect of sleep hours on next‐day overall pain was not statistically significant.

Racial differences were identified at the within‐person level between sleep duration and overall next‐day pain, with a significant interaction between sleep duration and race (*B* = −0.07, 95% CI: −0.12 to −0.01, *p *= 0.02; Table S3). Simple slope analysis revealed that, for Black caregivers, sleeping longer than own average was significantly associated with lower next‐day overall pain (*B* = −0.05, 95% CI: −0.09 to −0.01, *p* = 0.03), whereas for White individuals, the effect of sleep hours on next‐day overall pain was not statistically significant (*B* = 0.02, 95% CI: −0.02 to 0.06, *p* = 0.34; Figure 4). The above racial difference appeared to be driven by *evening pain*, where a significant interaction between sleep duration and race was identified (*B* = −0.13, 95% CI: −0.22 to −0.05, *p* = 0.003). Simple slope analysis revealed that sleeping longer than usual was significantly associated with lower evening pain among Black caregivers (*B* = −0.10, 95% CI: −0.17 to −0.04, *p* = 0.002) but this within‐person association was not significant for White caregivers (*B* = 0.03, 95% CI: −0.03 to 0.09, *p* = 0.30; Figure S2). In contrast, interaction terms for race were not significant for morning pain (*B* = −0.05, 95% CI: −0.11 to 0.01, *p* = 0.075) or afternoon pain (*B* = −0.04, 95% CI: − 0.14 to 0.05, *p* = 0.363).

No racial differences were found in the within‐person association between levels of sleep disturbance and next‐day pain, as the interaction between sleep disturbance and race was not significant for overall pain (*B* = −0.03, 95% CI: −0.10 to 0.04, *p* = 0.42), morning pain (*B* = −0.03, 95% CI: −0.11 to 0.05, *p* = 0.46), afternoon pain (*B *= −0.03, 95% CI: −0.15 to 0.09, *p *= 0.60), or evening pain (*B *= −0.03, 95% CI: −0.16 to 0.09, *p* = 0.58; see Table S4).

All between‐person associations between sleep (i.e., duration and sleep disturbances) and pain intensity (overall and time‐specific) were similar for Black and White caregivers (interaction terms with race all *p’*s > 0.05).

### 3.4 Sensitivity tests

A series of sensitivity tests were conducted to assess the robustness of the study's main findings. We tested for a curvilinear association between sleep duration and pain by including a quadratic term to determine whether there is an optimal number of sleep hours on daily pain. However, this quadratic term was non‐significant across models (findings available upon request), suggesting that a linear model sufficiently captured the association in our data. We also examined sleep hours as a binary variable reflecting recommended sleep duration (0 = outside 6–9 h, 1 = within 6–9 h). Findings were similar, except that Black and White caregivers had comparable likelihoods of achieving the recommended daily sleep duration (odds ratio [OR] = 0.64; 95% CI: 0.33−1.25, *p* = 0.19; results available upon request).

We also examined whether better sleep (i.e., longer sleep duration and lower levels of sleep disturbance) predicted reduced fluctuations in pain across the day by using the SD of day‐level pain scores in place of the mean pain scores of each day. Within‐person associations were non‐significant: sleep duration was not associated with SD of pain (*B* = −0.001, *p* = 0.90), and neither was the SD of levels of sleep disturbance (*B* = −0.01, *p* = 0.44; findings available upon request).

To assess the possibility that daily pain may influence sleep that night, we examined the bidirectional effect of pain on same‐day (that night's) sleep. No within‐person effects of pain (overall, morning, afternoon, or evening) on sleep duration or levels of sleep disturbance were found, in contrast to our findings showing that greater levels of sleep disturbance predicted next‐day morning pain. However, between‐person analyses indicated that caregivers who experienced higher pain levels across the study period (overall, morning, afternoon, or evening) had fewer sleep hours and greater levels of sleep disturbances than those with lower pain levels (findings available upon request). Racial differences were non‐significant for both within‐ and between‐person effects.

## 4 DISCUSSION

This study advances the literature by moving beyond cross‐sectional designs and chronic pain populations to investigate two critical public health challenges among dementia caregivers: sleep problems and pain. Using EMA to measure sleep across several days and pain multiple times per day allows us to capture real‐world processes, which is especially important for caregivers whose pain may be closely tied to daily routines. This provides a temporally nuanced understanding of the daily association between sleep and pain in the dementia caregiving context. This study achieved a low attrition rate, indicating strong retention and low missingness in key measures.

Dementia caregivers in our sample reported an average of 6.51 h of sleep per night, which is about half an hour shorter than the National Sleep Foundation's recommended minimum of 7 h per night.^47^ This finding was consistent with a systematic review and meta‐analysis of 35 studies, which estimated that dementia caregivers lose between 2.4 and 3.5 h of sleep per week compared with age‐matched non‐caregivers.^3^ A qualitative study found that one of the most frequently set goals among dementia caregivers is to improve their own sleep.^48^ Indeed, such sleep deficits may accumulate over time, leading to chronic sleep conditions and health consequences across multiple health domains. For instance, daily diary research found that longer sleep duration before high‐stress days can buffer against stress and reduce anxiety among dementia caregivers,^17^ highlighting the importance of promoting adequate amounts of sleep as a protective resource in caregiving contexts.

Supporting our hypothesis, despite reporting similar levels of sleep disturbances, Black caregivers reported 0.38 fewer hours of sleep (≈23 min) per night than White caregivers. Prior quantitative and qualitative studies have identified structural, socioeconomic, and environmental challenges (e.g., racism‐related vigilance, chronic stress, shift work, long hours, multiple jobs, housing instability, neighborhood noise, and safety concerns),^28^, ^29^, ^49^ as factors contributing to shorter sleep duration among Black individuals. Indeed, our descriptive data indicate that Black caregivers were more likely to provide nighttime care, which may also contribute to reduced overall sleep time. Although prior studies have also shown that Black individuals generally report poorer sleep quality beyond shorter sleep hours^27^, ^28^, ^29^; paradoxically, our findings challenge this trend by revealing no differences in sleep disturbances between Black and White dementia caregivers. Using National Study of Caregiving (NSOC) data, Osakwe and colleagues^50^ also found no differences in sleep interruptions between Black and White dementia caregivers, but Black caregivers were less likely than White caregivers to report difficulty falling back asleep. These paradoxical findings suggest that racial differences in dementia caregiving contexts could be more complex and warrant further examination.

In this sample of dementia caregivers, mean daily pain ratings were below 1 on a 0–5 scale, indicating no to very mild pain. This finding is notable for two reasons. First, it suggests that this group of caregivers may be relatively healthy, with minimal physical limitations in providing and sustaining care. Second, the low levels of pain should be considered when interpreting the lack of certain within‐person associations between sleep and pain in the current analyses. Nonetheless, despite these low pain levels in our sample, contrary to our hypothesis, Black caregivers reported significantly lower pain intensity than White caregivers. Indeed, a study using NSOC data showed that Black caregivers report lower rates of arthritis and activity‐limiting pain than White caregivers.^51^ Several potential explanations may account for this pattern, including strengths‐based perspective such as greater resilience and coping mechanisms (e.g., positive appraisal), as well as differences in expectations and pain reporting. For instance, Black caregivers may have developed resilience and adaptive coping strategies over time due to repeated exposure to adversity, which could lead to better regulation in response to physical discomfort.^52^, ^53^, ^54^ In addition, Black caregivers often have a more positive appraisal of caregiving, perceiving it as less stressful and more rewarding than White caregivers.^55^ As a result, pain may be perceived differently in this caregiving context, with Black caregivers reporting it primarily when severe, whereas White caregivers may be more likely to report moderate levels of discomfort. This speculation is further supported by population‐based studies showing that Black individuals report lower prevalence of less severe pain but higher prevalence of severe pain than White individuals.^32^, ^56^ These explanations provide a possible account for our findings; however, research on racial differences in pain in the dementia caregiver population remains limited; further studies are needed to confirm the mechanisms behind these differences.

Our full sample showed that when dementia caregivers had greater levels of sleep disturbances than usual the night before, they reported greater pain in the morning, suggesting that short‐term sleep disruptions (e.g., waking up too early, feeling unrested) may “sensitize” caregivers to pain, particularly upon waking. This finding aligns with results from experimental studies that have shown that sleep disruption and disturbances can provoke a spontaneous increase in pain perception and hyperalgesia, related particularly in females to attenuation of descending pain inhibition in the central nervous system.^57^ More importantly, our results reveal that caregivers who, on average, slept longer and experienced fewer disturbances reported lower overall pain levels than those who slept less and experienced more disturbances, whereas nightly deviations from their usual sleep patterns appeared to have less impact on next‐day pain. These distinctions suggest that chronic sleep patterns (between‐person effects), rather than short‐term variability (within‐person effects) may be more critical for pain management.

Finally, our finding that longer sleep duration was linked to reduced next‐day pain only among Black caregivers, but not White caregivers, supports our hypothesis. Coherent with existing literature, chronic stress from macro‐level inequalities (e.g., socioeconomic disadvantage, cumulative social stressors, and environmental exposures),^58^, ^59^, ^60^, ^61^, ^62^, ^63^, ^64^, ^65^ may not only reduce the opportunity to sleep, but may also be linked to more pronounced physiological responses in Black individuals (e.g., increased inflammation and cortisol dysregulation),^34^, ^66^, ^67^, ^68^, ^69^ which may in turn be related to higher daily pain and other adverse health outcomes. These findings highlight the importance of addressing both sleep and race‐related stress to improve health outcomes among Black caregivers, as these factors may interact and reinforce one another in a cyclical pattern.

### 4.1 Limitations and future directions

This study has several limitations that warrant future research. First, regarding measurement, pain is a biopsychosocial experience influenced by psychosocial context, physical activity, caregiving tasks, injury, substance use, and medications. Among dementia caregivers, physical pain may be compounded by emotional distress from witnessing a loved one's decline and loss of close relationships,^70^ which future studies could capture. Sleep was assessed via self‐report; future research should use objective measures, such as actigraphy or wearable/nearable devices, to improve accuracy and detect subtle disturbances. Additional sleep features, including regularity (e.g., sleep variability), timing, efficiency, and excessive daytime sleepiness, should also be examined.^71^, ^72^ Data on PLWD sleep patterns, bedroom‐sharing arrangements, and caregivers’ pre‐caregiving sleep and pain were not collected, highlighting the need for dyad and longitudinal studies.^73^, ^74^

Second, this study did not define clinically meaningful changes in pain, either within a day or over time. Establishing these thresholds in future research would enable better assessment of sleep's short‐term effects on pain. Third, this study did not assess pain variability or pain‐related interference, which may be more relevant than pain intensity score alone for understanding daily fluctuations and functional impacts in dementia caregivers. Moreover, the mechanisms linking poor sleep to daily or chronic pain remain unclear. Future studies could investigate how factors such as stress (chronic stress, stress reactivity, caregiving‐related stress), fatigue, and mood may serve as pathways linking sleep and pain. Finally, our sample may be healthier (e.g., may have relatively better sleep and lower pain; healthier individuals may have self‐selected into the study) than dementia caregivers in general. Therefore, replication in larger samples with chronic sleep problems and higher levels of pain is needed to enhance generalizability.

In summary, our findings provide new insights into racial differences in sleep patterns, daily pain, and the temporal sleep−pain association among dementia caregivers. Although Black caregivers reported shorter sleep duration, their daily pain was lower than that of White caregivers. Among overall dementia caregivers, higher levels of sleep disturbances were associated with greater pain in the morning. Longer sleep duration may play a particularly important role in alleviating or regulating the short‐term pain among Black caregivers than White caregivers. Although we lack direct measures of chronic sleep patterns, the data suggest that typical sleep patterns over time, rather than daily variations, are more consistently linked to pain. These findings highlight the need for sleep‐based interventions (e.g., nighttime care services for care recipients) that promote regular, healthy sleep to support caregivers’ physical health. Ensuring adequate sleep may be particularly needed for alleviating pain among Black caregivers. Future research should further explore the biopsychosocial factors and mechanisms underlying these associations.

## CONFLICT OF INTEREST STATEMENT

The authors declare no conflicts of interest. Author disclosures are available in the supporting information.

## ETHICS STATEMENT

The study was approved by the University of Michigan Ethics Review Board and was performed in accordance with the ethical standards as laid down in the 1964 Declaration of Helsinki and its later amendments or comparable ethical standards.

## CONSENT STATEMENT

All human subjects provided written informed consent.

## STATEMENT ADDRESSING DIVERSITY, EQUITY AND INCLUSION (DEI)

This study not only recruited a diverse group of dementia caregivers, including Black and White participants to enhance generalizability, but also specifically examined racial differences in the research questions. It also included caregivers of individuals with dementia who had not received a formal diagnosis but exhibited sufficient signs of dementia, addressing the lower rates of dementia diagnoses among Black Americans.

## Supporting information




**Supporting Information**: alz71518‐sup‐0001‐SuppMat.docx

**Supporting Information**: alz71518‐sup‐0002‐SuppMat.pdf
