# Supplementary material for: Sleep and daily pain intensity among Black and White dementia caregivers
Source: Alzheimers Dement. 2026 May 26;22(5):e71518. doi: 10.1002/alz.71518 (PMC13239835; doi:10.1002/alz.71518)

|  | 1. | 2. | 3. | 4. | 5. | 6 | 7. | 8. | 9. | 10. | 11. | 12. | 13. |
| --- | --- | --- | --- | --- | --- | --- | --- | --- | --- | --- | --- | --- | --- |
| 1. Black | - |  |  |  |  |  |  |  |  |  |  |  |  |
| 1. Age | -.08 | - |  |  |  |  |  |  |  |  |  |  |  |
| 1. Female | .12 | -.13 | - |  |  |  |  |  |  |  |  |  |  |
| 1. Married | -.33*** | .42*** | -.10 | - |  |  |  |  |  |  |  |  |  |
| 1. College or above | -.07 | .13 | -.05 | .09 | - |  |  |  |  |  |  |  |  |
| 1. Work part/full-time | -.00 | -.30*** | -.00 | -.08 | .07 | - |  |  |  |  |  |  |  |
| 1. Depression | .11 | -.36*** | .16* | -.21** | -.05 | .15* | - |  |  |  |  |  |  |
| 1. Anxiety | .11 | -.34*** | .21** | -.19** | -.06 | .12 | .68*** | - |  |  |  |  |  |
| 1. BMI | .19** | -.18* | .13 | -.09 | -.15* | .04 | .23*** | .18** | - |  |  |  |  |
| 1. Spousal caregivers | -.37*** | .57*** | -.10 | .77*** | .09 | -.13 | -.19** | -.17* | -.12 | - |  |  |  |
| 1. Adult child caregivers | .32*** | -.41*** | .10 | -.61*** | .00 | .07 | .13 | .18* | .16* | -.78*** | - |  |  |
| 1. Other caregivers | .11 | -.27*** | .02 | -.29*** | -.14* | .10 | .10 | .00 | -.06 | -.39*** | -.27*** | - |  |
| 1. Caregiver burden sum | -.04 | -.16* | .24*** | -.09 | .14* | .07 | .55*** | .53*** | .07 | -.13 | .21** | -.10 | - |
| 1. Help duration (in years) | .06 | .08 | .05 | -.06 | .05 | -.03 | .01 | .02 | -.04 | -.05 | .09 | -.06 | .12 |
| 1. Sleep hours | -.24*** | .24*** | .05 | .28*** | .21** | -.08 | -.28*** | -.22** | -.05 | .26*** | -.25*** | -.03 | -.08 |
| 1. Sleep disturbances | .01 | -.32*** | -.01 | -.24*** | -.18* | .11 | .50*** | .36*** | .07 | -.25*** | .18* | .12 | .28*** |
| 1. Nighttime caregiving | .14* | -.05 | .07 | -.13 | -.17* | -.03 | .04 | -.02 | .02 | -.07 | .03 | .05 | -.04 |
| 1. Overall daily pain | -.07 | -.18* | .11 | -.01 | -.20** | -.14* | .27*** | .13 | .22** | -.07 | .07 | .02 | .12 |
| 1. Morning pain | -.07 | -.15* | .11 | .01 | -.15* | -.14 | .24*** | .09 | .23*** | -.04 | .04 | .00 | .12 |
| 1. Afternoon pain | -.06 | -.18** | .09 | -.03 | -.24*** | -.15* | .26*** | .15* | .21** | -.09 | .08 | .03 | .11 |
| 1. Evening pain | -.08 | -.21** | .10 | -.00 | -.21** | -.14 | .31*** | .19** | .24*** | -.08 | .07 | .02 | .13 |

**Supplementary Table 1.**

Bivariate correlations at participant-level

Notes: **p* <.05; ***p* <.01; *** *p*<.001.

**Supplementary Table 1.**

Bivariate correlations at participant-level (continued)

|  | 14. | 15. | 16. | 17. | 18. | 19. | 20. |
| --- | --- | --- | --- | --- | --- | --- | --- |
| 15. Sleep hours | -.05 | - |  |  |  |  |  |
| 1. Sleep disturbances | .05 | -.48*** | - |  |  |  |  |
| 1. Nighttime caregiving (binary) | .21** | -.33*** | .21** | - |  |  |  |
| 1. Overall daily pain | -.00 | -.23*** | .31*** | .08 | - |  |  |
| 1. Morning pain | -.00 | -.21** | .29*** | .08 | .98*** | - |  |
| 1. Afternoon pain | -.02 | -.26*** | .30*** | .06 | .98*** | .92*** | - |
| 1. Evening pain | .02 | -.25*** | .32*** | .12 | .96*** | .91*** | .95*** |

Notes: ***p* <.01; *** *p*<.001

**Supplementary Table 2.**

Bivariate correlations at day-level

|  | 1. | 2. | 3. | 4. | 5. | 6. |
| --- | --- | --- | --- | --- | --- | --- |
| 1. Sleep hours | - |  |  |  |  |  |
| 2. Sleep disturbances | -.44*** | - |  |  |  |  |
| 3. Nighttime caregiving (binary) | -.26*** | .21*** | - |  |  |  |
| 4. Overall daily pain | -.17*** | .24*** | .05 | - |  |  |
| 5. Morning pain | -.14*** | .23*** | .04 | .96*** | - |  |
| 6. Afternoon pain | -.18*** | .22*** | .04 | .95*** | .83*** | - |
| 7. Evening pain | -.15*** | .22*** | .03 | .90*** | .78*** | .85*** |

Notes: *** *p*<.001.

**Supplementary Table 3.**

Multilevel Linear Models Predicting Overall Pain and Pain in Different Times of the Day From Sleep Disturbances: Moderated by Race

|  | Overall Pain | | |  | Morning Pain | | |  | Afternoon Pain | | |  | Evening Pain | | |
| --- | --- | --- | --- | --- | --- | --- | --- | --- | --- | --- | --- | --- | --- | --- | --- |
|  | B  (SE) | 95% CI | p-value |  | B  (SE) | 95%  CI | p-value |  | B  (SE) | 95%  CI | p-value |  | B  (SE) | 95%  CI | p-value |
| Intercept | 1.79 (0.77) | [0.28, 3.31] | .020 |  | 1.65 (0.74) | [0.19, 3.10] | .027 |  | 2.64 (0.85) | [0.98, 4.30] | .002 |  | 1.87 (0.88) | [0.15, 3.59] | .033 |
| Black | -0.55 (0.66) | [-1.84, 0.75] | .408 |  | -0.32 (0.63) | [-1.56, 0.92] | .610 |  | -1.17 (0.75) | [-2.64, 0.30] | .119 |  | -0.46 (0.80) | [-2.02, 1.10] | .560 |
| **Within-person association** | | | | | | | | | | | | | | | |
| Sleep hours | 0.02 (0.02) | [-0.02, 0.06] | .337 |  | 0.02 (0.02) | [-0.03, 0.07] | .419 |  | 0.00 (0.03) | [-0.05, 0.05] | .926 |  | 0.03 (0.03) | [-0.03, 0.09] | .304 |
| Sleep hours × Black | **-0.07 (0.03)** | **[-0.12, -0.01]** | **.022** |  | -0.05 (0.03) | [-0.11, 0.01] | .075 |  | -0.04 (0.05) | [-0.14, 0.05] | .361 |  | -0.13 (0.05) | [-0.22, -0.05] | .003 |
| **Between-person association** | | | | | | | | | | | | | | | |
| Sleep hours | -0.17 (0.07) | [-0.31, -0.03] | .016 |  | -0.15 (0.07) | [-0.29, -0.01] | .031 |  | -0.26 (0.08) | [-0.42, -0.11] | .001 |  | -0.17 (0.08) | [-0.33, -0.01] | .041 |
| Sleep hours × Black | 0.03 (0.10) | [-0.16, 0.23] | .752 |  | 0.00 (0.10) | [-0.18, 0.19] | .972 |  | 0.13 (0.11) | [-0.10, 0.35] | .265 |  | 0.00 (0.12) | [-0.24, 0.24] | .979 |
| Participant-level covariates | | | | | | | | | | | | | | | |
| Age | -0.01 (0.01) | [-0.02, 0.01] | .303 |  | -0.01 (0.01) | [-0.02, 0.01] | .335 |  | -0.01 (0.01) | [-0.02, 0.01] | .325 |  | -0.01 (0.01) | [-0.02, 0.00] | .187 |
| Female | 0.19 (0.15) | [-0.10, 0.48] | .191 |  | 0.20 (0.15) | [-0.08, 0.49] | .164 |  | 0.19 (0.16) | [-0.11, 0.50] | .219 |  | 0.16 (0.16) | [-0.14, 0.47] | .301 |
| Married | 0.35 (0.20) | [-0.04, 0.75] | .081 |  | 0.30 (0.19) | [-0.09, 0.68] | .128 |  | 0.30 (0.21) | [-0.11, 0.71] | .154 |  | 0.49 (0.23) | [0.05, 0.94] | .028 |
| College or above | -0.22 (0.13) | [-0.48, 0.03] | .087 |  | -0.16 (0.13) | [-0.41, 0.09] | .217 |  | -0.35 (0.14) | [-0.62, -0.07] | .013 |  | -0.23 (0.15) | [-0.52, 0.07] | .130 |
| Work part/full-time | -0.39 (0.12) | [-0.63, -0.16] | .001 |  | -0.36 (0.12) | [-0.59, -0.14] | .002 |  | -0.37 (0.13) | [-0.63, -0.12] | .004 |  | -0.49 (0.14) | [-0.76, -0.22] | .<000 |
| Depression | 0.05 (0.02) | [0.01, 0.08] | .005 |  | 0.04 (0.02) | [0.01, 0.08] | .010 |  | 0.05 (0.02) | [0.01, 0.08] | .008 |  | 0.06 (0.02) | [0.03, 0.10] | .001 |
| Anxiety | -0.20 (0.12) | [-0.44, 0.04] | .107 |  | -0.25 (0.12) | [-0.49, -0.01] | .041 |  | -0.18 (0.13) | [-0.45, 0.08] | .168 |  | -0.15 (0.14) | [-0.42, 0.13] | .297 |
| BMI | 0.02 (0.01) | [0.00, 0.04] | .026 |  | 0.02 (0.01) | [0.00, 0.04] | .016 |  | 0.02 (0.01) | [0.00, 0.04] | .090 |  | 0.02 (0.01) | [0.00, 0.04] | .046 |
| Spouse caregivers |  |  |  |  |  |  |  |  |  |  |  |  |  |  |  |
| Adult child caregivers | 0.25 (0.22) | [-0.19, 0.68] | .269 |  | 0.15 (0.22) | [-0.28, 0.58] | .499 |  | 0.23 (0.23) | [-0.21, 0.68] | .297 |  | 0.43 (0.25) | [-0.05, 0.92] | .082 |
| Other caregivers | 0.22 (0.30) | [-0.36, 0.81] | .456 |  | 0.13 (0.28) | [-0.42, 0.69] | .637 |  | 0.22 (0.32) | [-0.40, 0.85] | .480 |  | 0.34 (0.35) | [-0.34, 1.02] | .324 |
| Caregiver burden | 0.00 (0.01) | [-0.02, 0.02] | .919 |  | 0.00 (0.01) | [-0.02, 0.02] | .765 |  | 0.00 (0.01) | [-0.02, 0.02] | .954 |  | -0.01 (0.01) | [-0.03, 0.01] | .511 |
| Help duration | 0.00 (0.01) | [-0.02, 0.03] | .880 |  | 0.00 (0.01) | [-0.02, 0.02] | .961 |  | 0.00 (0.01) | [-0.03, 0.03] | .840 |  | 0.01 (0.02) | [-0.03, 0.04] | .716 |
| Day-level covariates | | | | | | | | | | | | | | | |
| Nighttime caregiving (WP) | 0.01 (0.05) | [-0.09, 0.11] | .861 |  | -0.02 (0.05) | [-0.11, 0.08] | .726 |  | 0.09 (0.07) | [-0.05, 0.23] | .202 |  | 0.00 (0.11) | [-0.23, 0.22] | .969 |
| Nighttime caregiving (BP) | -0.01 (0.18) | [-0.36, 0.34] | .962 |  | 0.01 (0.18) | [-0.34, 0.35] | .974 |  | -0.13 (0.19) | [-0.50, 0.25] | .507 |  | 0.00 (0.21) | [-0.41, 0.41] | .993 |
| Observations |  | 854 |  |  |  | 854 |  |  |  | 830 |  |  |  | 789 |  |
| # participants |  | 209 |  |  |  | 209 |  |  |  | 205 |  |  |  | 203 |  |

**Supplementary Table 4.**

Multilevel Linear Models Predicting Overall Pain and Pain in Different Times of the Day From Sleep Disturbances Moderated by Race

|  | Overall Pain | | |  | Morning Pain | | |  | Afternoon Pain | | |  | Evening Pain | | |
| --- | --- | --- | --- | --- | --- | --- | --- | --- | --- | --- | --- | --- | --- | --- | --- |
|  | B  (SE) | 95% CI | p-value |  | B  (SE) | 95%  CI | p-value |  | B  (SE) | 95%  CI | p-value |  | B  (SE) | 95%  CI | p-value |
| Intercept | 0.43 (0.64) | [-0.82, 1.68] | .497 |  | 0.45 (0.61) | [-0.73, 1.64] | .454 |  | 0.56 (0.69) | [-0.80, 1.92] | .418 |  | 0.60 (0.72) | [-0.80, 2.01] | .400 |
| Black | -0.24 (0.19) | [-0.61, 0.13] | .209 |  | -0.23 (0.19) | [-0.61, 0.15] | .236 |  | -0.12 (0.21) | [-0.53, 0.28] | .554 |  | -0.40 (0.20) | [-0.79, -0.02] | .041 |
| **Within-person association** | | | | | | | | | | | | | | | |
| Sleep disturbances | 0.03 (0.02) | [-0.01, 0.08] | .161 |  | 0.06 (0.03) | [0.01, 0.12] | .029 |  | 0.01 (0.03) | [-0.06, 0.07] | .786 |  | -0.01 (0.04) | [-0.09, 0.07] | .760 |
| Sleep disturbances × Black | -0.03 (0.04) | [-0.10, 0.04] | .416 |  | -0.03 (0.04) | [-0.11, 0.05] | .463 |  | -0.03 (0.06) | [-0.15, 0.09] | .600 |  | -0.03 (0.06) | [-0.16, 0.09] | .578 |
| **Between-person association** | | | | | | | | | | | | | | | |
| Sleep disturbances | 0.21 (0.09) | [0.04, 0.38] | .017 |  | 0.20 (0.09) | [0.04, 0.37] | .016 |  | 0.26 (0.09) | [0.08, 0.45] | .005 |  | 0.22 (0.10) | [0.02, 0.42] | .027 |
| Sleep disturbances × Black | 0.00 (0.12) | [-0.24, 0.23] | .969 |  | 0.02 (0.12) | [-0.21, 0.26] | .835 |  | -0.10 (0.13) | [-0.36, 0.16] | .447 |  | 0.06 (0.14) | [-0.21, 0.33] | .668 |
| Participant-level covariates | | | | | | | | | | | | | | | |
| Age | -0.01 (0.01) | [-0.02, 0.01] | .338 |  | -0.01 (0.01) | [-0.02, 0.01] | .343 |  | -0.01 (0.01) | [-0.02, 0.01] | .411 |  | -0.01 (0.01) | [-0.02, 0.00] | .191 |
| Female | 0.18 (0.15) | [-0.10, 0.47] | .209 |  | 0.21 (0.14) | [-0.08, 0.49] | .152 |  | 0.16 (0.16) | [-0.14, 0.47] | .295 |  | 0.15 (0.15) | [-0.16, 0.45] | .343 |
| Married | 0.34 (0.20) | [-0.05, 0.73] | .086 |  | 0.29 (0.19) | [-0.09, 0.66] | .137 |  | 0.29 (0.21) | [-0.12, 0.70] | .166 |  | 0.47 (0.22) | [0.03, 0.91] | .035 |
| College or above | -0.22 (0.13) | [-0.47, 0.03] | .080 |  | -0.14 (0.12) | [-0.38, 0.10] | .245 |  | -0.36 (0.14) | [-0.63, -0.08] | .011 |  | -0.22 (0.15) | [-0.50, 0.07] | .139 |
| Work part/full-time | -0.38 (0.12) | [-0.62, -0.15] | .001 |  | -0.35 (0.12) | [-0.58, -0.13] | .002 |  | -0.35 (0.13) | [-0.61, -0.09] | .009 |  | -0.49 (0.14) | [-0.76, -0.21] | <.001 |
| Depression | 0.04 (0.02) | [0.01, 0.08] | .023 |  | 0.03 (0.02) | [0.00, 0.07] | .053 |  | 0.04 (0.02) | [0.01, 0.08] | .024 |  | 0.05 (0.02) | [0.01, 0.09] | .011 |
| Anxiety | -0.19 (0.12) | [-0.43, 0.05] | .116 |  | -0.24 (0.12) | [-0.48, -0.01] | .041 |  | -0.18 (0.13) | [-0.44, 0.08] | .182 |  | -0.15 (0.14) | [-0.42, 0.12] | .281 |
| BMI | 0.02 (0.01) | [0.00, 0.04] | .023 |  | 0.02 (0.01) | [0.00, 0.04] | .012 |  | 0.02 (0.01) | [0.00, 0.04] | .083 |  | 0.02 (0.01) | [0.00, 0.04] | .035 |
| Spouse caregivers |  |  |  |  |  |  |  |  |  |  |  |  |  |  |  |
| Adult child caregivers | 0.26 (0.22) | [-0.17, 0.69] | .241 |  | 0.15 (0.21) | [-0.27, 0.57] | .492 |  | 0.27 (0.22) | [-0.18, 0.71] | .238 |  | 0.44 (0.25) | [-0.05, 0.92] | .078 |
| Other caregivers | 0.16 (0.30) | [-0.42, 0.74] | .580 |  | 0.07 (0.28) | [-0.48, 0.61] | .814 |  | 0.18 (0.32) | [-0.44, 0.80] | .574 |  | 0.25 (0.34) | [-0.42, 0.92] | .463 |
| Caregiver burden | 0.00 (0.01) | [-0.02, 0.02] | .857 |  | 0.00 (0.01) | [-0.02, 0.02] | .927 |  | 0.00 (0.01) | [-0.02, 0.02] | .770 |  | -0.01 (0.01) | [-0.03, 0.01] | .363 |
| Help duration | 0.00 (0.01) | [-0.02, 0.02] | .987 |  | 0.00 (0.01) | [-0.02, 0.02] | .920 |  | 0.00 (0.01) | [-0.03, 0.03] | .906 |  | 0.00 (0.02) | [-0.03, 0.03] | .827 |
| Day-level covariates | | | | | | | | | | | | | | | |
| Nighttime caregiving (WP) | 0.00 (0.05) | [-0.10, 0.11] | .946 |  | -0.03 (0.05) | [-0.13, 0.07] | .544 |  | 0.10 (0.07) | [-0.05, 0.24] | .183 |  | 0.01 (0.12) | [-0.22, 0.23] | .954 |
| Nighttime caregiving (BP) | 0.04 (0.19) | [-0.33, 0.41] | .834 |  | 0.04 (0.18) | [-0.32, 0.40] | .836 |  | -0.03 (0.21) | [-0.44, 0.37] | .874 |  | 0.02 (0.22) | [-0.41, 0.45] | .929 |
| Observations |  | 855 |  |  |  | 855 |  |  |  | 831 |  |  |  | 790 |  |
| # participants |  | 210 |  |  |  | 210 |  |  |  | 206 |  |  |  | 204 |  |

**Supplementary Figure 1. Mean pain intensity at different time points of the day by race.**

**
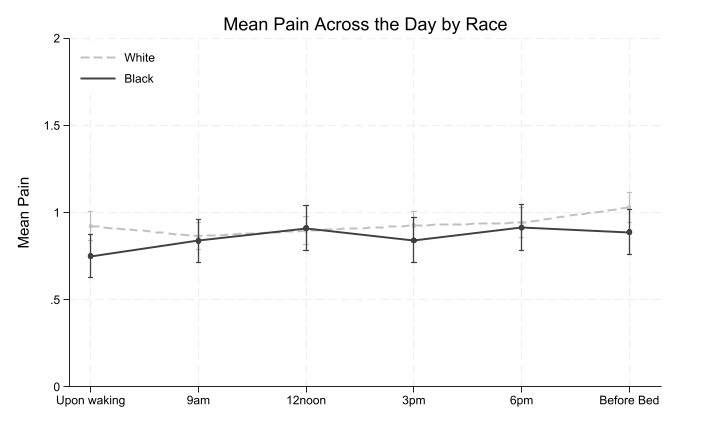
**

**Supplementary Figure 2. Within-person links between sleep duration and evening pain.** Simple slope analysis revealed that sleeping longer than usual was significantly associated with lower evening pain among Black caregivers, but this within-person association was not significant for White caregivers.


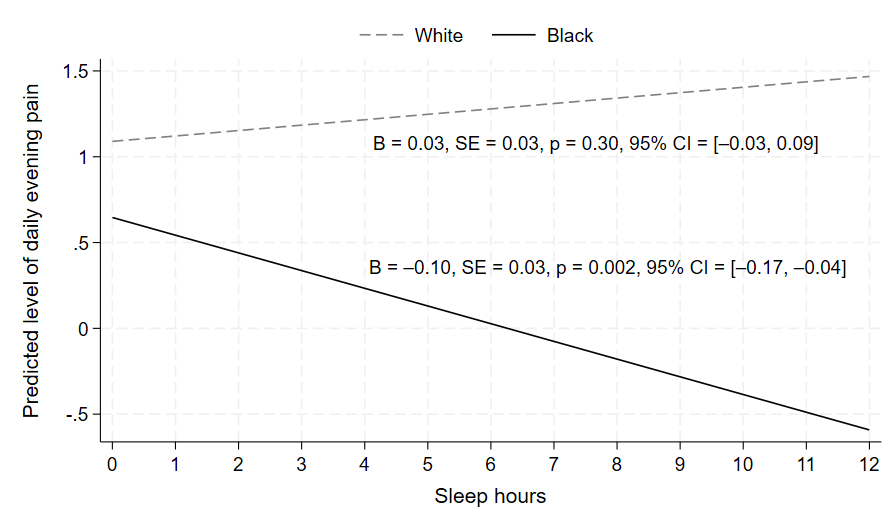

Supplement: Supplementary file 1 — Supporting Information: alz71518‐sup‐0001‐SuppMat.docx [file ALZ-22-e71518-s001.docx]
